# A cross-sectional study to compare care needs of individuals with and without dementia in residential homes in the Netherlands

**DOI:** 10.1186/1471-2318-13-51

**Published:** 2013-05-24

**Authors:** Eva S van der Ploeg, Dieuwertje Bax, Marijke Boorsma, Giel Nijpels, Hein PJ van Hout

**Affiliations:** 1 Department of Public Health, Erasmus Medical Centre, Rotterdam, the Netherlands; 2 Aged Mental Health Research Unit, Monash University, Cheltenham, VIC, Australia; 3 Department of General Practice and Old Age Medicine, VU University Medical Centre Amsterdam, Institute for Research in Extramural Medicine (EMGO+), Amsterdam, the Netherlands

**Keywords:** Dementia, Needs assessment, Residential care, CANE

## Abstract

**Background:**

Little is known about met and unmet needs of individuals in residential care, many of whom suffer from dementia. Unmet needs are associated with a decreased quality of life, worse mental health, dissatisfaction with services, and increased costs of care. The aim of this study was to compare the number and type of (unmet) needs of people with and without dementia in residential care in the Netherlands.

**Methods:**

187 individuals in residents care or their relatives were interviewed to identify their care needs on 24 topics using the Camberwell Assessment of Needs for the Elderly (CANE) interview.

**Results:**

Individuals diagnosed with probable dementia reported more needs in total and more unmet needs in comparison with individuals without this diagnosis. More specifically, differences were found for the topics “accommodation”, “money”, “benefits”, “medication management”, “incontinence”, “memory problems”, “inadvertent self-harm”, “company” and “daytime activities”.

**Conclusions:**

It seems that the differences in care needs between individuals with and without dementia can be attributed to actual differences in physical and cognitive functioning. Residents with dementia reported more often unmet needs which might imply that care for people with dementia can still be better attuned to their needs.

## Background

In the Netherlands and other developed countries the population is ageing, in 2007 14% of the population was 65 years or older, but this is estimated to increase to 24% in 2050 [1]. Not only do more people grow old, but on average they also live longer. This results in an increase of individuals with limited daily functioning and consequently in higher institutionalisation rates. The number of people with dementia will also increase significantly. Almost 6% of people aged 65 and over have dementia and this percentage increases exponentially with age to 40% amongst people aged 90 and over [2,3].

Individuals in residential care are mostly older, vulnerable and in need of both assistance with activities of daily living (ADL) and sheltered living because of their physical and cognitive impairments. Residential care facilities in the Netherlands do not provide specialised medical and nursing care; the general practitioner is responsible for the medical care. Of the older persons living in residential homes in the Netherlands it is estimated that 25% suffers from dementia [4]. This percentage is expected to increase exponentially as a consequence of the aging of the population.

Dementia is a neuropsychiatric disorder characterized by memory problems and cognitive impairment (e.g. aphasia and apraxia), that has a significant negative impact on social or professional functioning and represents a decline compared to the previous level of functioning [5]. The disorder has many disabling consequences, including potentially underreporting care needs, because of difficulties in communication. Care needs are often defined as a state where help (or more help) with specific difficulties is required according to the person who expresses the need [6]. When an individual receives no help or perceives the received help as insufficient or inappropriate the need is considered to be unmet. Unmet needs are more prevalent in individuals with dementia and are known to lead to decreased quality of life and higher levels of distress in this group [7,8]. Therefore, care providers should better attune care to individual needs of people with dementia. To deliver appropriate care, unmet needs should be identified [9]. Many studies have focussed on showing differences between the care needs that individuals with dementia report compared to what their caregivers report. The results of these studies were mixed. One study found high agreement on reported number and type of needs between people with dementia and their staff and relatives as well as high agreement on reported service use [10]. Other studies found that people with dementia reported fewer needs (both met and unmet) compared to staff and relatives [11-14]. It is not clear whether these differences are due to underreporting of the individuals with dementia or to over-concerned staff and relatives. A recent study in community-dwelling people with dementia in the Netherlands revealed that they reported most unmet needs for the domains of memory, information, company, psychological distress and daytime activities [13]. A narrative review of the literature on needs of people with dementia and their caregivers across settings reported that the most important needs included a need for information, support in regards to the symptoms of dementia, social contact and company and for health monitoring and safety [14].

To our knowledge, no comparisons have been made between the care needs reported by individuals with and without dementia in residential settings. Since both groups have been admitted to residential care and are in need of high care, we will explore if the needs, both met and unmet, differ. This may assist staff and family to become more aware of and more responsive to the needs of people residing in long term care.

The aim of this study was to describe the difference in number and type of care needs of people with and without dementia living in residential care. For a subgroup of the people with severe dementia a proxy reported their needs as they themselves were no longer able. We hypothesise that the number of met and unmet needs will be lowest in the group with no diagnosis of dementia, followed by the group with dementia who were still able to self-report with the most reported needs for the group with dementia and proxy-reports.

## Methods

### Design

This cross-sectional study was conducted in ten residential care facilities in a north-western region of the Netherlands. The study was approved by the VU Medical Centre Ethics Committee.

### Research sample

All residential care facilities (n = 10) from one care organisation in the north-west of the Netherlands were included in this study, including both rural and urban areas. This care organisation displayed an interest in participating in this project with the purpose of improving their quality of care.

**Figure 1:**
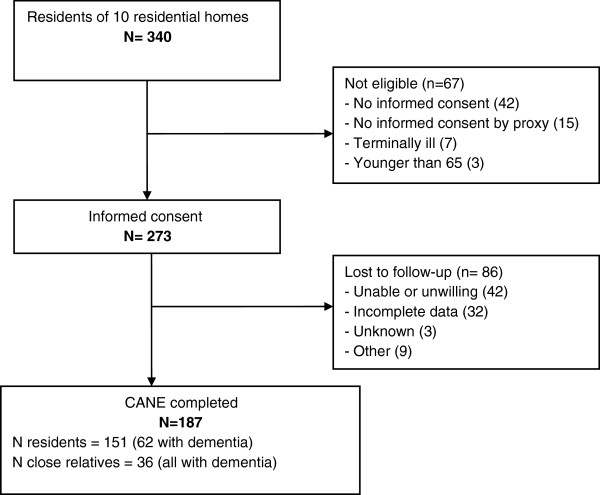
Flow chart of selection of residents and proxies.

All residents of these facilities (n = 340) were screened for eligibility to participate. Terminally ill residents as determined by staff or primary care physician and residents under 65 years of age were excluded. All residents had to sign informed consent and in the case of decisional incompetence a close relative was asked to sign informed consent. We tried to limit the burden for the resident by dividing the baseline measurement over two appointments on separate days to be able to collect all the information. The CANE was administered at the second appointment, which was scheduled within two weeks after the first appointment. If staff reported severe cognitive impairment, a close relative was asked to provide consent and a proxy report (n = 57). 67 individuals were not eligible: 57 residents or relatives did not consent to the study, 7 residents were terminally ill and 3 were younger than 65 (Figure 1).

Of the 273 eligible residents 86 persons were lost to follow-up: 42 individuals were unable or unwilling to participate, 32 were not able to complete all the measures (e.g. refrained from participating in the second part of the baseline interview) and 12 were lost for other reasons, of which 3 unknown. Thus, 36 interviews were completed by a close relative and 151 by residents themselves resulting in information on 187 persons in total.

### Measures & measurements

#### Dementia

The presence or absence of dementia was based on medical record information, crosschecked with cognitive testing. Dementia diagnoses were usually made by geriatricians or memory clinics. The Dutch clinical guidelines for diagnosing probable dementia refer to the DSM-IV criteria [5]. Diagnosis were crosschecked by administrating the 7-minute screen [15]. A cut of point of a ≥70% risk of dementia was used to distinguish between individuals with and without dementia. This criterion corresponds with a positive and negative predictive value of 91% and 96% respectively [16] and sensitivity and specificity of 93% and 94% [17].

#### Severity of dementia

To establish the severity of dementia, trained residential care staff scored cognition of the residents using the Cognitive Performance Scale (CPS) [18]. This concise scale is part of the Resident Assessment Instrument Long Term Care Facility (interRAI-LTCF) assessment, which was administered before the CANE-interview [19,20]. The CPS ranges between 0–7 and correlates strongly with scores generated by the Mini-Mental State Examination [18]. CPS-Scores of 2 and higher indicate cognitive impairment.

#### Care needs

The Camberwell Assessment of Need in the Elderly (CANE) is a structured interview to identify self-perceived care needs. The CANE consists of 24 topics in four care domains (Environmental, Physical, Psychological and Social needs) [12]. Examples of environmental needs are having a suitable living environment and being able to perform domestic tasks. Physical needs include diagnosed physical ailments as well as functional problems such as managing medication and being mobile. Psychological needs include experiencing difficulties with memory, mood and behaviour. Examples of social needs are experiencing a lack of company or more precise an intimate relationship.

The CANE has good content, construct and consensual validity and it demonstrates appropriate criterion validity. Reliability is generally very high with Kappa over 0.85 for staff ratings of interrater reliability in a study describing older people with mental disorders [12]. This instrument has been translated for use in the Netherlands and this version has shown acceptable construct and criterion validity for use in people with dementia and their proxies [16]. The test-retest reliability in this study was moderate to good for the majority of the CANE items (average Kappa of 0.60) [16].

The CANE-interviews took about 30 to 45 minutes to complete and were conducted by trained interviewers. The training consisted of a two day program before the study commenced. A manual was provided holding general and specific instructions of the study and its measurements. General attitude, interview techniques and the interview forms were explained and practised. During the study 2-monthly supervision meetings were held with the interviewers to discuss difficulties.

We recorded met, unmet and total needs. A met need was recorded when the resident identified a problem but felt there was appropriate help to significantly reduce the need. An unmet need was recorded when the resident identified a problem for which there was insufficient or no help [21]. The total number of needs was the sum of met and unmet needs for each topic.

#### Disability

The Groningen Activity Restriction Scale (GARS) is an easy-to-administer, comprehensive, reliable, hierarchical, and valid measure for assessing disability in Activities of Daily Living (ADL), Instrumental Activities of Daily Living (IADL) and mobility in aged populations. An overall score of the 18 items was calculated (range:18–72) [22]. Trained interviewers administered the GARS at the time of the CANE interview.

#### Demographics

Birth date, marital status and gender were recorded at the start of the CANE interview.

### Data analysis

We performed analysis using SPSS 14.0 for Windows. For the description of demographic information and number of care needs we used descriptive analyses and chi-square tests. Differences in needs between the three groups were analyzed using binary logistic regression with the non-dementia group as the reference group.

## Results

### Demographic and clinical characteristics

187 residents participated in the study. 94 participants had a diagnosis of dementia according to medical files (n = 90) or cognitive screening (n = 4); for 36 people with dementia we relied on proxy-reports. Hence we included 93 participants with no reported diagnosis of dementia; 58 people with dementia who reported needs themselves, and; 36 people with dementia for whom a proxy reported needs.

Approximately three quarters of the total study sample was female. Over two thirds of residents were widowed, 19% were married, 2% were divorced and 8% had never married at all (not in table). The mean age of participants was 87 years (range 72–98). The three study groups did not differ in age, gender and marital status (Table 1). However, they did differ in (I)ADL functioning and cognitive functioning. Bonferroni post-hoc testing showed that the non-dementia group had better (I)ADL and cognitive functioning than the two dementia groups (residents and relatives). The two dementia groups did not differ from one another in respect to (I)ADL and cognitive functioning.

**Table 1:** Characteristics of residents without dementia (n = 93) and for residents with dementia who completed the CANE themselves (n = 58) or for whom a proxi completed the CANE (n = 36)

		**No dementia**	**Dementia**		
			**Resident**	**Carer**	**p**
Female	n(%)	69 (76)	43 (72)	25 (69)	
Married	n(%)	19 (21)	9 (15)	7 (19)	
Age	M (SD)	85.8 (5.1)	86.7 (5.4)	87.3 (4.3)	
(I)ADL functioning (18–72)†	M (SD)	45.9 (11.5)^ab^	40.0 (13.3)^a^	36.5 (12.6)^b^	***
CPS-score (0–7) ††	M (SD)	.8 (1.2)^ab^	2.8 (1.8)^a^	3.0 (1.2)^b^	***

*** p < .001.

† Higher score means better functioning.

†† Higher score means worse cognition.

^ab^ Groups with the same letter differ significantly from one another.

### Differences in total needs

**Figure 2:**
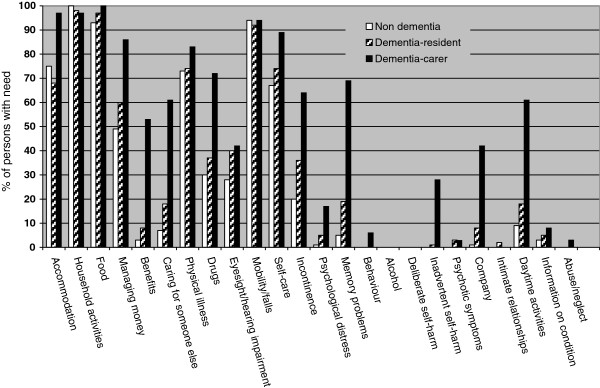
Percentages of residents without dementia (n = 89) and for residents with dementia who completed the CANE themselves (n = 62) or for whom a carer completed the CANE (n = 36) describing reported needs for each topic and in total.

The average number of needs in the total study sample was 7.8 (SD 2.6, range 3–16) and an average of 0.4 (SD 0.94, range 0–7) needs was unmet (not in table). Relatives of individuals with dementia reported more needs in total and for the environmental, physical and social domains than both people who did not have dementia and those with dementia who were able to complete the CANE themselves (Table 2). People without dementia reported less unmet needs than both dementia groups. The total number of psychological needs showed a stepwise pattern with people without dementia reporting the least needs, people with dementia who could complete the CANE reported more needs and people with dementia who could not complete the CANE reported the most needs (Figure 2).

**Table 2:** Average needs in total and per care domain for residents without dementia (n = 93) and for residents with dementia who completed the CANE themselves (n = 58) or for whom a proxi completed the CANE (n = 36)

		**No dementia**	**Dementia**		
	**Range**	**M (SD)**	**Resident M (SD)**	**Carer M (SD)**	**p**
Total needs	0-24	6.46 (1.41)^ab^	7.11 (1.97)^b^	10.33(2.69)^ab^	***
Total unmet needs	0-24	.14 (.47)^ab^	.39 (1.04)^a^	.83 (1.44)^b^	**
Total environmental needs	0-6	3.28 (.77)^a^	3.34 (.83)^b^	4.36 (.68)^ab^	***
Total physical needs	0-6	3.13 (1.10)^a^	3.53 (1.40)^b^	4.44 (1.50)^ab^	***
Total psychological needs	0-7	.06 (.23)^ab^	.29 (.55)^ac^	1.22 (.90)^bc^	***
Total social needs	0-5	.13 (.34)^a^	.32 (.76)^b^	1.14 (.83)^ab^	***

**p < .01, *** p < .001.

^abc^ Groups with the same letter differ significantly from one another.

### Differences in need topics

#### Environmental needs

Using logistic regression analyses with the non-dementia group as reference group showed that the three groups significantly differed in the number of needs reported for “accommodation”, “money” and “benefits” (Table 3). Individuals without dementia reported the least number of needs, people with dementia who were able to complete the CANE reported more needs and people with dementia for whom proxy reports were obtained reported the highest number of needs on support in living, managing finances and obtaining benefits. No differences were found for “household activities” and “food”; for which almost everyone reported a need. The differences for “caring for someone” were not significant.

**Table 3:** Number of residents or relatives that reported a need for each care topic

	**Persons without dementia (n = 93)**	**Persons with dementia - Resident completed CANE (n = 58)**		**Persons with dementia – Proxy completed CANE(n = 36)**	
	**n (%)**	**n (%)**	**OR (95%CI)**	**n (%)**	**OR (95%CI)**
			**(Ref. category = No dementia)**		**(Ref. category = Resident)**
*Environmental needs*					
Accommodation	67 (75)	42 (68)	.69 (.34-1.41)	35 (97)	16.67 (2.13-130.49)**
Household activities	89 (100)	61 (98)	-	35 (97)	.57 (.04-9.46)
Food	83 (93)	60 (97)	2.17 (.42-11.12)	36 (100)	-
Managing money	44 (49)	37 (60)	1.51 (.79-2.92)	31 (86)	4.19 (1.43-12.24)**
Benefits	3 (3)	5 (8)	1.76 (.56-5.52)	19 (53)	11.00 (3.93-30.76)***
Caring for someone else	6 (7)	11 (18)	.46 (.09-2.36)	22 (61)	.86 (.08-9.80)
*Physical needs*					
Physical illness	65 (73)	46 (74)	1.06 (.51-2.22)	30 (83)	1.74 (.61-4.95)
Drugs	27 (30)	23 (37)	1.35 (.68-2.69)	26 (72)	4.41 (1.81-10.77)**
Eyesight/hearing impairment	25 (28)	25 (40)	1.73 (.87-3.44)	15 (42)	1.06 (.46-2.44)
Mobility/falls	84 (94)	57 (92)	.68 (.19-2.45)	34 (94)	1.49 (.27-8.11)
Self-care	60 (67)	46 (74)	1.39 (.68-2.86)	32 (89)	2.78 (.85-9.10)
Incontinence	18 (20)	22 (36)	2.17 (1.04-4.52)*	23 (64)	3.22 (1.37-7.57)**
*Psychological needs*					
Psychological distress	1 (1)	3 (5)	4.47 (.45-44.06)	6 (17)	3.93 (.92-16.83)
Memory problems	4 (5)	12 (19)	5.10 (1.56-16.67)**	25 (69)	9.47 (3.67-24.45)***
Behaviour	0 (0)	0 (0)	-	2 (6)	-
Alcohol	0 (0)	0 (0)	-	0 (0)	-
Deliberate self-harm	0 (0)	0 (0)	-	0 (0)	-
Inadvertent self-harm	0 (0)	1 (1)	-	10 (28)	23.46 (2.86-192.79)**
Psychotic symptoms	0 (0)	2 (3)	-	1 (3)	.86 (.08-9.80)
*Social needs*					
Company	1 (1)	5 (8)	7.72 (.88-67.79)	15 (42)	8.14 (2.63-25.18)***
Intimate relationships	0 (0)	1 (2)	-	0 (0)	-
Daytime activities	8 (9)	11 (18)	2.18 (.82-5.79)	22 (61)	7.29 (2.86-18.55)***
Information on condition	3 (3)	3 (5)	1.46 (.28-7.47)	3 (8)	1.79 (.34-9.37)
Abuse/neglect	0 (0)	0 (0)	-	1 (3)	

*p < .05,**p < .01, *** p < .001.

#### Physical needs

The three groups differed significantly in the number of needs reported for “medication(management)” and “incontinence”. Individuals without dementia reported the least number of needs, people with dementia who completed the CANE themselves reported more needs and the people with dementia and proxy reports reported the highest number of needs. No differences were found on needs concerning “physical illness” and “mobility/falls”: over three quarters reported needs for these topics. People without dementia reported slightly less difficulties with eyesight and hearing, but this difference was not significant.

#### Psychological needs

The three groups differed in the number of needs reported for “memory” and “inadvertent self-harm” (e.g. wandering and leaving candles burning). Again the pattern was stepwise as described above. Hardly anyone reported needs for “behavioural problems”, “alcohol (abuse)”, “deliberate self-harm” and “psychotic symptoms”.

#### Social needs

The three groups reported different numbers of needs for the topics “need for company” and “daytime activities” (sufficient things to do to get through the day). Hardly anyone without dementia had problems with these needs, some of the individuals who had dementia but were able to complete the CANE reported these needs and a quite substantial number of the people with dementia and a proxy report, reported these needs. Hardly anyone reported needs for “having an intimate relationship”, “information on condition” and “abuse/neglect”.

## Discussion

The aim of this study was to describe the difference in number and type of care needs of persons with and without dementia living in residential care. We compared the total number of needs and unmet needs regarding 24 need topics. Our hypothesis was partly confirmed: people diagnosed with dementia reported more total needs and unmet needs than people without dementia, and within the dementia group proxy’s reported more needs than residents themselves. More specifically, differences were found for the topics “accommodation”, “money”, “benefits”, “medication management”, “incontinence”, “memory problems”, “inadvertent self-harm”, “company” and “daytime activities”. It seems that the differences in care needs between the dementia and the non-dementia group can be attributed to actual differences in physical and cognitive functioning, whereas within the dementia group the people who reported the needs (resident or proxy) seemed to be the most important factor. Some need patterns of persons with and without dementia met our expectations (such as a difference on “memory problems” but similar scores on “physical illness”).

### Strengths

This is the first study to report on differences in needs between persons with and without dementia living in residential care. Since the response rate and number of residents in this study was relatively high, the results may well reflect the actual number of needs and unmet needs in residential care in the Netherlands despite the fact that participating facilities where located in one geographic area. Our findings can support residential staff and family members to become more aware of and responsive to the needs of people with and without dementia.

### Limitations

It is possible that people with the most (unmet) needs and/or most advanced dementia were under recruited, because they were unable to participate. The study sample may have been less disabled than the general population of residents. Thus our findings may have underestimated the number of (unmet) needs. We have partly tried to address this limitation by including proxy reports for some residents.

Another limitation of the study might be social desirability, i.e. the tendency to answer in ways that people believe others find acceptable and approve of [23]. For example, although people with dementia did report more memory needs, only half of the individuals with a diagnosis of dementia reported a memory need, which seems low if one is diagnosed with this condition. Another explanation for this finding may include that people with dementia who do not report a need for memory problems may not be aware of any memory problems.

### Comparison to other studies

Although, previous studies described the needs of people with dementia, to our knowledge, there are no studies that compare the needs of persons with and without dementia in residential homes. In line with some studies [11-14] relatives reported more needs than residents themselves, whereas residents and relatives reported similar rates of objective physical and cognitive functioning. A number of reasons could underlie this difference: people with dementia may not recall having certain needs or may have forgotten some of the services that have been provided. Alternatively, they may not want to complain or they may not be aware of the services that they can ask for. On the other hand, families may overestimate the needs of their relative with dementia or may not be aware of the services delivered.

Furthermore, the number of needs and unmet needs of persons with dementia in this study was generally lower (an average of 7.8 in total and 0.4 unmet needs) in comparison with other needs assessment studies on people with dementia in residential care [10,11,24]. This comparatively low count of needs may be due to the fact that the number of needs in some of the other studies was based on the reported needs by resident, staff and sometimes the carer together whereas we only used proxy reports for the most disabled persons. In general, relatives and staff report more needs than people with dementia themselves. It is also possible that our Dutch population was less disabled than the population of the other studies, which were conducted in the UK. Admittance criteria for residential care vary across countries and these may be stricter in the UK compared to the Netherlands, thereby selecting a group with more needs when studying the residential care population. Therefore, generalisability of the number of reported needs to other countries may be limited, although the actual differences between the groups may still apply. However, some need topics seem to be prevalent across settings and countries, e.g. needs for company and information [13,14].

The number of unmet needs in this sample was low. Possibly, the care in the participating residential care facilities was responsive to individuals’ expressed needs. On the other hand, it might be possible that older persons based their needs on the care they thought was available. However, people with dementia reported more unmet needs than people without dementia which might imply that care for people with dementia can still be better attuned to their needs.

## Conclusions

This first study to explore care needs in the residential care setting emphasises the importance of carefully establishing care needs for people with dementia. Relatives reported more needs than people with dementia themselves and this difference was not accounted for by actual differences in physical and cognitive functioning. Staff in residential care may want to consider discussing care needs with both the individual themselves and their families and integrate these reports with their own impression of the individual. The low number of unmet needs amongst people with no diagnosis of dementia may be due to appropriate care provided by the residential care facilities or a tendency of residents to attune their needs to the available resources.

## Competing interests

The authors declare that they have no competing interests.

## Authors’ contributions

DB and EvdP were involved in data collection, analysed the data and drafted the paper. MB, GN, HvH designed the study. HvH supervised the data collection. All authors read and approved the final manuscript.

## Pre-publication history

The pre-publication history for this paper can be accessed here:

http://www.biomedcentral.com/1471-2318/13/51/prepub
